# Natural course of chronic fatigue syndrome/myalgic encephalomyelitis in adolescents

**DOI:** 10.1136/archdischild-2016-311198

**Published:** 2017-01-19

**Authors:** Tom Norris, Simon M Collin, Kate Tilling, Roberto Nuevo, Stephen A Stansfeld, Jonathan AC Sterne, Jon Heron, Esther Crawley

**Affiliations:** 1 Centre for Child and Adolescent Health, University of Bristol, Bristol, UK; 2 School of Social & Community Medicine, University of Bristol, Bristol, UK; 3 CIBER Epidemiología y Salud Pública (CIBERESP), Instituto de Salud Carlos III, Madrid, Spain; 4 Wolfson Institute of Preventive Medicine, Queen Mary University of London, UK

**Keywords:** Chronic Fatigue Syndrome, CFS/ME, ALSPAC, Adolescent Health, Longitudinal

## Abstract

**Objective:**

Little is known about persistence of or recovery from chronic fatigue syndrome/myalgic encephalomyelitis (CFS/ME) in adolescents. Previous studies have small sample sizes, short follow-up or have focused on fatigue rather than CFS/ME or, equivalently, chronic fatigue, which is disabling. This work aimed to describe the epidemiology and natural course of CFS/ME in adolescents aged 13–18 years.

**Design:**

Longitudinal follow-up of adolescents enrolled in the Avon Longitudinal Study of Parents and Children.

**Setting:**

Avon, UK.

**Participants:**

We identified adolescents who had disabling fatigue of >6 months duration without a known cause at ages 13, 16 and 18 years. We use the term ‘chronic disabling fatigue’ (CDF) because CFS/ME was not verified by clinical diagnosis. We used multiple imputation to obtain unbiased estimates of prevalence and persistence.

**Results:**

The estimated prevalence of CDF was 1.47% (95% CI 1.05% to 1.89%) at age 13, 2.22% (1.67% to 2.78%) at age 16 and 2.99% (2.24% to 3.75%) at age 18. Among adolescents with CDF of 6 months duration at 13 years 75.3% (64.0% to 86.6%) were not classified as such at age 16. Similar change was observed between 16 and 18 years (75.0% (62.8% to 87.2%)). Of those with CDF at age 13, 8.02% (0.61% to 15.4%) presented with CDF throughout the duration of adolescence.

**Conclusions:**

The prevalence of CDF lasting 6 months or longer (a proxy for clinically diagnosed CFS/ME) increases from 13 to 18 years. However, persistent CDF is rare in adolescents, with approximately 75% recovering after 2–3 years.

**What is already known on this topic?**

- Chronic fatigue syndrome/myalgic encephalomyelitis (CFS/ME) is relatively common and disabling in children and adolescents. Previous studies (typically with small samples) have reported inconsistent estimates of persistence of CFS/ME during adolescence. The long-term prognosis of the condition in those not receiving treatment is not known.

**What this study adds?**

- The prevalence of chronic disabling fatigue (CDF), a proxy for clinically diagnosed CFS/ME, increases during adolescence. Approximately 25% persist over a 2–3-year follow-up. Only 8% of children with CDF at age 13 had CDF at 16 and 18 years.

## Introduction

Chronic fatigue syndrome/myalgic encephalomyelitis (CFS/ME) is relatively common (prevalence 0.5–3%) and disabling in children and adolescents.^1–4^ The disease has been defined by various criteria.^5^

In the UK, National Institute for Health and Care Excellence (NICE) guidelines state that diagnosis of CFS/ME should be made in children after 3 months of persistent or recurrent fatigue, which is not the result of ongoing exertion, not substantially alleviated by rest, has resulted in a substantial reduction in activities, is characterised by post-exertional malaise, the presence of at least one recognised symptom and has no known cause.^6^ The most commonly used research criteria (Centers for Disease Control and Prevention (CDC)) require 6 months duration of fatigue, which has significantly interfered with daily activities, and the individual must have four or more recognised symptoms of CFS/ME.^7^

CFS/ME has a significant impact on an adolescent's education, family and social life. Children attending specialist (secondary care) services attend, on average, 2 days a week of school.^8^ Two thirds are unable to attend school at all when the condition is at its most severe, with an average total absence from school of 1 year (at 4 years follow-up).^9^ ^10^ Most children (96%) stop socialising with their friends^10^ and there are emotional and financial consequences for families.^11^ Understanding the prognosis of this condition in children and adolescents is therefore vital. However, little is known about the persistence of, or recovery from, CFS/ME in adolescence.

A systematic review of observational studies reported that 50–94% of adolescents made a good or complete recovery at 13–72 months.^12^ However, the sample sizes included in this review were small (median=78; range=15–498), the duration of symptoms at outset and length of follow-up were highly variable, and it only included participants accessing secondary or tertiary care. More recently, a small number of observational studies have been conducted, which followed adolescents with CFS/ME recruited into specialist services, that revealed less favourable recovery rates of 40–70%.^9^ ^13^ ^14^ ^15^ It is therefore difficult to say with any certainty what proportion of adolescents who do not receive treatment will recover and what proportion will have persistent fatigue. Furthermore, the extent to which CFS/ME in early adolescence increases the risk of developing CFS/ME in later adolescence is unknown.

The aim of this study was to describe the prevalence of CFS/ME at 18 years and the persistence and recovery of CFS/ME during adolescence. By defining persistent fatigue using a 6 months criterion, we aimed to compare persistence and recovery consistent with the widely used CDC diagnostic criteria for CFS/ME.^7^ ^16^ However, results based on a 3-month criterion of fatigue duration, in line with the NICE diagnostic criteria, will also be presented in the online supplementary material. As children in our study were not examined by a physician, we have used the term ‘chronic disabling fatigue’ (CDF) rather than CFS/ME to indicate chronic fatigue that is disabling.

10.1136/archdischild-2016-311198.supp1 supplementary material

## Methods

### Sample

**Figure 1:**
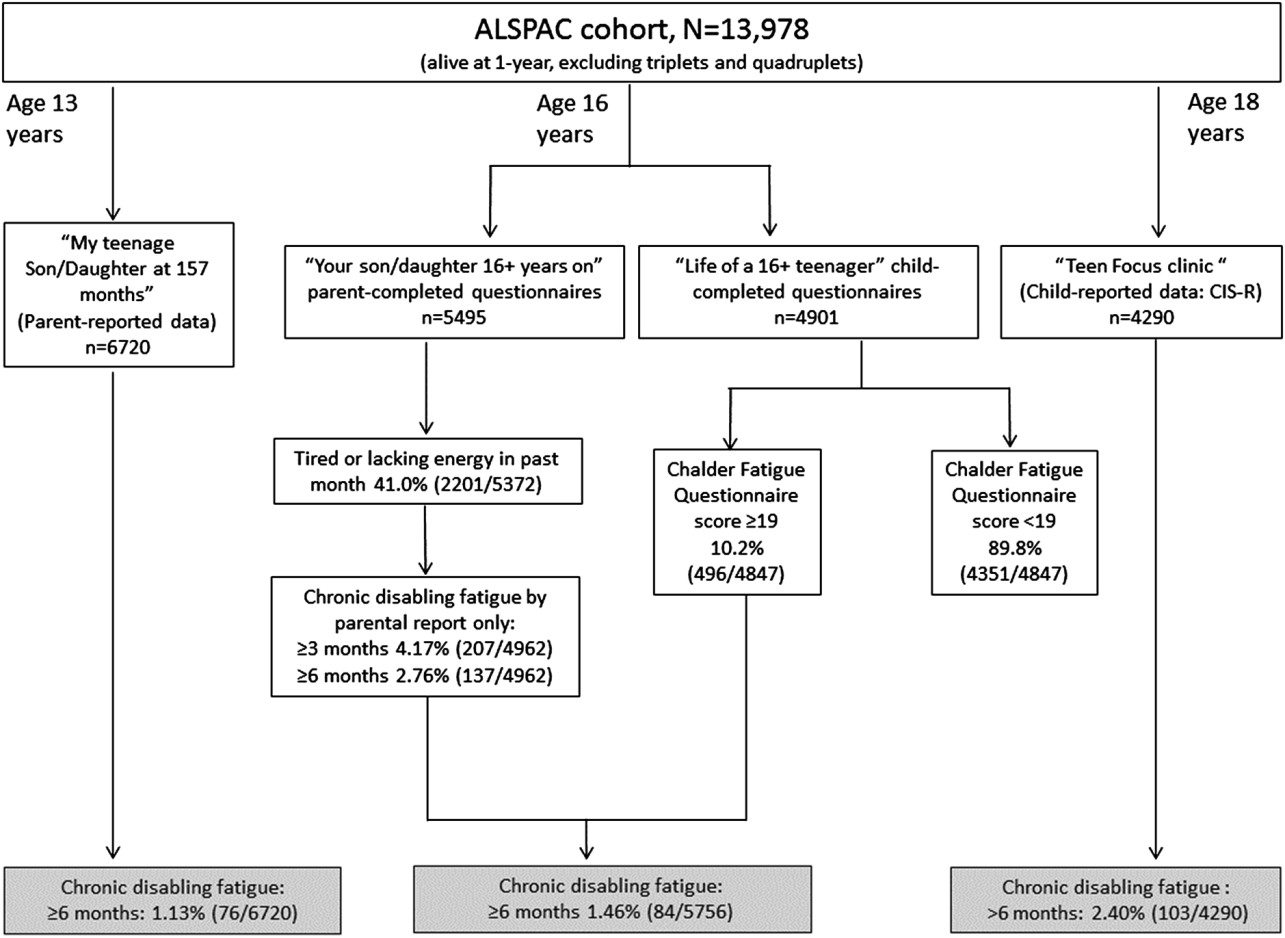
Flowchart showing number of subjects with available information in each age and those with chronic disabling fatigue. ALSPAC, Avon Longitudinal Study of Parents and Children.

The Avon Longitudinal Study of Parents and Children (ALSPAC) is a population-based longitudinal birth cohort of children who had an expected date of delivery between April 1991 and December 1992 and whose mothers were resident in the Avon region of Southwest England at the time of recruitment.^17^ From 14 541 pregnancies included, 13 978 children were alive at 12 months of age (excluding triplets and quadruplets). The children have been followed up regularly since birth with postal questionnaires for both children and their parents, clinical assessments and the collection of biological samples (please note that the study website contains details of all the data that is available through a fully searchable data dictionary: http://www.bris.ac.uk/alspac/researchers/data-access/data-dictionary/). Figure 1 shows the available data at each time point and the number of cases classified with CDF.

### Variables

#### CDF at age 13 years

Our method for defining CDF at age 13 years has been described previously.^1^ In brief, we identified adolescents reported by their mothers to have experienced fatigue lasting >6 months that was associated with absence from full-time school or that had prevented them from taking part in activities ‘quite a lot’ or ‘a great deal’. We excluded those whose mothers thought that the fatigue was caused by playing too much sport, who snored often and who had other illnesses that could cause fatigue (based on self-reported medication use). Adolescents were assigned a missing value for CDF if the question for school attendance had not been answered.

#### CDF at age 16 years

Our method for defining CDF at age 16 years has been described previously.^18^ Briefly, a classification of CDF was based on both parental and child-reported data. We classified adolescents as chronically fatigued if parents had reported that they had fatigue lasting >6 months that had stopped them from taking part in activities ‘quite a lot’ or ‘a great deal’, that was not due to playing too much sport and that had resulted in any absence from school/college in the past year due to tiredness or lack of energy. The child reported data that was used in order to classify CDF was based on the Chalder Fatigue Questionnaire,^19^ with a score of ≥19 (out of 33) representing CDF. A cut-off score of 19/33 has a sensitivity of 82.4% and a specificity of 86.4% for CFS/ME in adolescence.^20^ Teenagers were classified as non-CDF if the parent had provided answers indicating CDF but the Chalder score was <19. Furthermore, teenagers were classified as having CDF if they met the criteria by parental report but Chalder fatigue data were missing, under the assumption that teenagers with CDF would be less likely to have completed the ‘Life of a 16+ teenager’ questionnaire.

#### CDF at age 18 years

At age 17/18 (median 17.8, IQR 17.6 to 17.9) years, adolescents attended a ‘Focus @ 17’ clinic, at which they completed a computer-based Revised Clinical Interview Schedule (CIS-R).^21^ Participants were classified as CDF if they indicated that they had been getting tired or had been lacking in energy during the past month and then responded ‘yes' to >2 of the following four items: (1) felt tired or lacking in energy for 4 days or more in the past seven days; (2) felt tired or lacking in energy for more than 3 hours in total on any day in the past seven days; (3) felt so tired or lacking in energy that they had to push themselves to get things done on one or more occasion in the past seven days and (4) felt tired or lacking in energy when doing things they enjoy in the past seven days.

We classified the following as not CDF: the tiredness or lack of energy had lasted for <6 months; the adolescent thought it was due to exercise or medication; the adolescent felt better after resting or if exercise did not make them feel exhausted the following day. The CIS-R also provided data on seven of the eight symptoms of CFS/ME specified in CDC diagnostic criteria,^7^ namely muscle or joint pain, headaches, painful glands, sore throat, problems with memory or concentration (cognitive dysfunction) and insomnia (as a proxy for ‘unrefreshing sleep’). We required the presence of at least one of these symptoms, rather than the minimum of four required by CDC criteria, as this is consistent with UK guidance for the diagnosis of CFS/ME. Adolescents were classified as not having CDF if they reported having had problems with alcohol or drugs (crack, solvents, heroin or cocaine) during the previous year or a diagnosis of anorexia nervosa.

At all three time points, the presence of comorbid depressive symptoms was not an exclusion criteria for the classification of CDF, but sensitivity analyses excluding those with significant depressive symptoms (≥11 on the Short Moods and Feelings questionnaire (SMFQ))^22^ were conducted. The SMFQ correlates well with other measures of depression and has good test–retest reliability.^23^ ^24^ A cut-off score of ≥11 was used to indicate high levels of depressive symptoms, a threshold that has shown high sensitivity, specificity and negative predictive power for an International Classification of Disease-10 diagnosis of depression at age 18 years in this cohort.^25^

### Statistical analysis

First, the crude prevalence of CDF, using a criterion of at least 6 months, was estimated at each time point among those adolescents for whom sufficient data were available to define its presence or absence.

#### Recovery/persistence from CDF

Subjects with CDF at one time point (age 13 or age 16) were categorised as having persistent CDF across two time points (13–16 or 16–18) if they were also classified as having CDF at the next time point, otherwise classified as recovered.

#### Multiple imputation to address missing data

Performing only complete-case analyses (ie, ignoring those adolescents with missing values for CDF or covariates) could result in bias and would result in inflation of SEs. If missingness is dependent only on observed data (referred to as missing at random), then multiple imputation can be used to correct this bias. We generated 90 imputed data sets based on a set of variables that were selected because of either their strong hypothesised association with CDF, the relatedness to the missingness of CDF and/or the amount of missing data in these variables themselves (considered in order to construct stable imputation models that would produce reliable estimates). These included fatigue (measured at 13, 16 and 18 years), maternal age at delivery, maternal education, total family adversity index (FAI) score recorded during pregnancy (representing multiple indicators of family, parental and sociodemographic risk), total FAI score recorded when the child was 8–10 years old, three additional components of the FAI recorded at 8–10 years, total authorised absences during year 11 of schooling, total unauthorised absences during year 11 of schooling, mean Key Stage 2 mark and total score on the Strength and Difficulties Questionnaire measured when the child was 11 (descriptions of these are provided in the online supplementary material). The number of imputations required to achieve convergence of parameter estimates was determined by checking the estimate of the Monte Carlo error (MCerror) in relation to the SE of the coefficient being estimated. In particular, the number of imputations was increased incrementally and when the MCerror achieved a value that was <10% of the SE of the estimate, the number of imputations was deemed adequate. The sample after imputation was 13 978, which represents those who were in the ‘core’ ALSPAC sample, who were alive at 1 year and who were either a singleton or twin. Multivariable imputation was performed using the univariate chained equations method paired with regression switching^26^ (using Stata's user-written ‘ice’ command), combining estimates using Rubin's rules.^27^

Whereas the prevalence of CDF at 18 was calculated in the both the complete case and imputed data sets, the persistence and recovery will only report on the imputed estimates, to provide less biased estimates of the persistence and recovery (complete case results included in the online supplementary material). Imputed estimates were rounded to the nearest integer to aid interpretability. Analyses were performed using Stata V.13.1 (StataCorp. 2013. College Station, Texas, USA: StataCorp LP).

## Results

Data necessary for a classification of CDF were available for 6720 adolescents at 13 years, 5756 at 16 years and 4290 at 18 years. Table 1 shows that those with missing fatigue data were more likely to be male, have a higher FAI (at 8–10 years), have depressive symptoms at 18.6 years and were less likely to apply to university.

**Table 1:** Descriptive statistics for those with chronic disabling fatigue (CDF) at 18, those without CDF and those with missing data

	Data on fatigue (n=4290)				Missing fatigue data (n=9688)	p Value‡
	CDF (n=103)	Not CDF (n=4187)	p Value*†	Total		
Sex (male)	27/103 (26%)	1850/4187 (44.2%)	<0.001	1877/4290 (43.8%)	5343/9688 (55.2%)	<0.001
FAI at 8–10 years (range 0–14)	1.39 (1.06–1.71)	0.98 (0.94–1.03)	0.005	0.99 (0.95–1.03)	1.11 (1.07–1.51)	<0.001
Number of AS levels obtained#	3.49 (3.09–3.88)	3.52 (3.46–3.58)	0.88	3.52 (3.46–3.58)	3.51 (3.41–3.60)	0.82
Number of A-levels studying#	2.69 (2.18–3.21)	2.83 (2.75–2.91)	0.54	2.83 (2.75–2.91)	2.85 (2.73–2.96)	0.76
Depression at 18 (yes)	20/50 (40%)	421/2158 (19.5%)	<0.001	441/2208 (20.0%)	248/985 (25.2%)	0.001
Applied to university (yes)	29/50 (58%)	1324/2158 (61.4%)	0.63	1353/2208 (61.3%)	490/990 (49.5%)	<0.001
Currently has a paid job (yes)	26/46 (57%)	1309/1955 (67.0%)	0.14	1335/2001 (66.7%)	563/880 (64.0%)	0.153
Number of CFS/ME symptoms (≥4) (CDC criteria)	30/103 (29%)	218/4187 (5.2%)	<0.001	248/4290 (5.8%)	–	–

*χ^2^ tests for proportions, t-tests for means.

†CDF versus not CDF. #AS level: Advanced Subsidiary Level taken at 17 years A level: General Certificate of Education Advanced Level taken at 18 years.

‡Fatigue data versus missing fatigue data.

CDC, Centers for Disease Control and Prevention; CFS/ME, chronic fatigue syndrome/myalgic encephalomyelitis; FAI, Family adversity index.

In those with enough data to make a classification of CDF, adolescents with CDF were more likely to be female, have higher levels of family adversity (at age 8–10) and more likely to have depressive symptoms at 18.6 years compared with those without CDF. Online supplementary table S1 displays the distribution of, and amount of data for, the imputed variables in those in the complete case analysis.

### Prevalence at 18

At age 18 years, 2.40% (103/4290) were classified as having CDF lasting 6 months or longer (95% CI 1.98 to 2.90), with 29.1% (95% CI 21.1% to 38.7%) of these fulfilling the CDC criteria (four or more symptoms, 6 months fatigue), while 100% fulfilled the NICE criteria of ≥1 symptom. The previously reported higher 16-year prevalence observed in females persisted at 18 (76/2413, 3.15%; 95% CI 2.52% to 3.93%) compared with males (n=27/1877, 1.44%; 95% CI 0.99% to 2.09% (difference=1.71%; 95% CI 0.83 to 2.59, p<0.001)). Imputed 18-year estimates revealed a prevalence of 2.99% (95% CI 2.24% to 3.75%), with the sex difference persisting (females: 3.58%; 95% CI 2.74 to 4.42%; males: 2.44%; 95% CI 1.25% to 3.63%).

Of those who had both CDF at 18 years and available depression data, 40% (20/50) had depressive symptoms. If all those with any depressive symptoms during adolescence (13, 16 or 18 years) were recoded to non-CDF, the prevalence of CDF at 18 was 1.21% (95% CI 0.92% to 1.59%). This increased to 2.17% after imputation.

Online supplementary table S2 shows the prevalence estimates for CDF at 13 and 16 years, using both the 3-month and 6-month criteria.

### Recovery from/persistence of CDF

**Figure 2:**
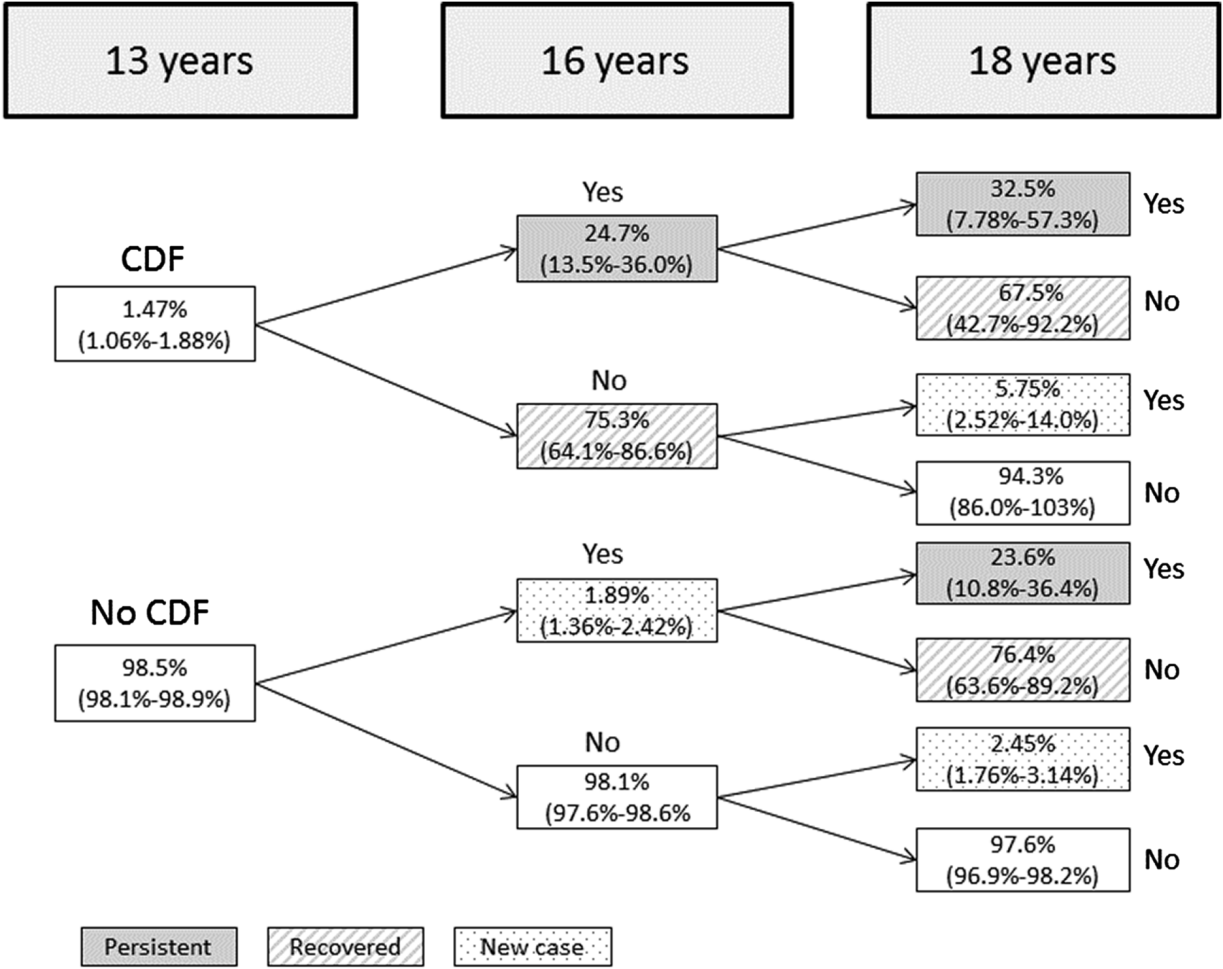
Proportion (SE) of those showing recovery from/persistence of chronic disabling fatigue (CDF) between 13 and 18 years old, after multiple imputation of missing data.

Between 13 and 16 years, imputation revealed that 24.7% (95% CI 13.5% to 36.0%) of those with CDF at 13 also had CDF at 16 years (figure 2). A small percentage of the adolescents who had CDF at 13 also had it at 18 but not 16 (5.75% (95% CI 2.52% to 14.0%)). Between 16 and 18 years, 25.0% (95% CI 12.8 to 37.2%) of those with CDF at 16 also had CDF at 18 years. Finally, 8.02% (95% CI 0.61% to 15.4%) of adolescents with CDF at 13 maintained this classification at all three time points (tables 2 and 3 show the presence of CDF at later time points by presence of earlier CDF).

**Table 2:** Presence of chronic disabling fatigue (CDF) in total sample by presence at previous time point

		Based on multiple imputation* n/total (%)	Imputed OR* (95% CI)
CDF at age 16	CDF at 13	51/206 (24.7%)	16.8 (8.4–33.8)
	No CDF at 13	260/13 772 (1.9%)	
CDF age 18	CDF at 16	78/311 (25.0%)	12.9 (6.4–26.1)
	No CDF at 16	340/13 667 (2.5%)	
	CDF at 13, CDF at 16	17/51 (33.3%)	7.9 (3.2–19.3)
	CDF at 13, no CDF at 16	9/155 (5.8%)	
	No CDF at 13, CDF at 16	61/260 (23.5%)	12.1 (5.7–25.6)
	No CDF at 13, no CDF at 16	331/13 512 (2.5%)	

*Imputations based on previous CDF, maternal age at delivery, maternal education, total family index score recorded during pregnancy, total family adversity index (FAI) score recorded when the child was 8–10 years old, three additional components of the FAI recorded at 8–10 years, total authorised absences during year 11 of schooling, total unauthorised absences during year 11 of schooling, mean Key Stage 2 mark and total score on the Strength and Difficulties Questionnaire measured when the child was 11.

**Table 3:** Presence of chronic disabling fatigue (CDF) in males and females by presence at previous time point

		Based on multiple imputation* n/total (%)	Imputed OR (95% CI)*
Boys			
CDF at age 16	CDF at 13	30/119 (25.2%)	19.7 (7.4–52.7)
	No CDF at 13	117/7101 (1.6%)	
CDF at age 18	CDF at 16	38/147 (25.9%)	17.1 (5.0–58.6)
	No CDF at 16	139/7073 (2.0%)	
Girls			
CDF at age 16	CDF at 13	21/86 (24.4%)	14.2 (5.0–39.8)
	No CDF at 13	143/6672 (2.1%)	
CDF at age 18	CDF at 16	40/165 (24.2%)	10.0 (4.3–23.4)
	No CDF at 16	202/6593 (3.1%)	

*Imputations based on previous CDF, maternal age at delivery, maternal education, total family index score recorded during pregnancy, total family adversity index (FAI) score recorded when the child was 8–10 years old, three additional components of the FAI recorded at 8–10 years, total authorised absences during year 11 of schooling, total unauthorised absences during year 11 of schooling, mean Key Stage 2 mark and total score on the Strength and Difficulties Questionnaire measured when the child was 11.

Sensitivity analyses reclassifying those with CDF and any depressive symptoms during adolescence to non-CDF suggested that persistence between 13 and 16 years was similar (13–16 years: 24.2%; 95% CI 12.7% and 35.7%), but with reduced persistence between 16 and 18 years (18.5%; 95% CI 7.53% and 29.6%), and between all three time points (5.71%; 95% CI 0.71% and 12.13%).

Online supplementary table S3 shows presence of CDF at later time points by the presence of earlier CDF, defined using the 3-month fatigue criterion.

## Discussion

We have shown that, in a large cohort of adolescents, the prevalence of CDF increased with advancing age, so that, using imputed results, 1.47% (95% CI 1.05% to 1.89%) of adolescents had the condition at 13, increasing to 2.22% (95% CI 1.67% to 2.78%) at 16 years and 2.99% (95% CI 2.24% to 3.75%) at 18 years. Approximately 25% of adolescents showed persistence of CDF at the subsequent time point, which was 2 (16–18) or 3 (13–16) years later. 8.02% of those classified as having the condition at 13 would still have the condition at 16 and 18, suggesting that they probably had CDF throughout adolescence.

### Comparison with other studies

The proportion of children in our study with CDF at age 13 that persisted at age 16 was 24.7% (95% CI 13.5% to 36.0%), with a similar figure also observed between 16 and 18 years (25.0%; 95% CI 12.8 to 37.2%). This is slightly lower than that described in observational studies on clinic populations. Smith *et al*^28^ observed that, in a sample of 15 adolescents, CFS/ME had either persisted or worsened in 46.7% (by self-report) at follow-up 12–32 months later. Rangel *et al*^9^ followed 25 adolescents for 46 months and observed no improvement, or worsening of symptoms, in 32%. Similar persistence was observed at 3–4 years follow-up by Gill *et al*^13^ (44%) and Sankey *et al*^14^ (33.3%). Bell *et al*^29^ conducted a follow-up study of 35 adolescents, with an average follow-up of 13 years (average age at illness onset: 12.1 years). Compared with the aforementioned studies, persistence after this extended period of follow-up was lower, with only approximately 20% reporting being either as or more ill than at the study onset. Our lower persistence may reflect less severe disease in the population compared with clinical cohorts. We observed a group of adolescents (8.02%; 95% CI 0.61% to 15.4%) who were classified as CDF at all three time points. These adolescents may represent a group who were experiencing a more severe form of the disease, characterised by increased persistence or recurrence of the disease.

Our finding that CDF is more common in females at age 18 is consistent with a number of other studies in adolescence.^2^ ^30–32^ Indeed in this cohort, 16-year-old females had a similarly increased risk (OR 1.64 (1.00 to 2.70)).^18^ However, this is different to 13 year olds and primary school children where the prevalence has been observed to be equal in males and females.^1^ ^31^ This might be because of changing cortisol profile in females,^33^ or hormonal sex differences that arise during puberty, for example, testosterone, which has been shown to help prevent muscle fatigue^34^ and thus could contribute to this sex difference.^35^ Gender differences in coping strategies may be implicated, with females reportedly displaying more negative coping strategies during adolescence,^36^ or females may experience a disproportionate amount of stressful events during adolescence.^37^

### Strengths and limitations

This is, to our knowledge, the largest study with the longest follow-up to have investigated the persistence and recovery of CDF in adolescence. Furthermore, as we have defined CDF using a 6-month criterion and adopted a number of exclusionary criteria used in the classification of CFS/ME, we have produced estimates that are comparable with the widely used CDC definition of CFS/ME, albeit with more relaxed criteria regarding the number of symptoms required to be present (one rather than four).

As is commonly observed in longitudinal studies, loss to follow-up was apparent in our study, with increasing amounts of missingness with advancing age. However, the use of multiple imputation enabled the analysis to be conducted on the maximal sample size and thus increase precision in the estimates and correct for potential bias in prevalence estimates caused by differential losses to follow-up. As imputed estimates were higher than those derived from complete data, this suggests that adolescents who were lost to follow-up were more likely to have risk factors that are positively associated with CDF. Imputations were based on variables that were predictive of the pattern of missingness in CDF, or predictive of CDF itself, but which did not have substantial amounts of missing data (<50%). This decision was made because prior results from models including variables that had substantial amounts of missing data (>70%) produced unstable estimates for the prevalence of CDF. However, it is acknowledged that this approach may have led to variables with strong hypothesised associations with CDF being omitted from the imputation models.

The main limitation of the study is that our classification is based on a combination of self-report of symptoms experienced by the adolescents and by parental report, with no diagnosis confirmed by a paediatrician. We therefore cannot rule out that other diagnoses could have been present that explained the fatigue. However, the exclusion criteria employed, including those on medication, those who reported having had problems with alcohol or drugs in the previous year or received a diagnosis of anorexia nervosa, means we are likely to have excluded such conditions.

Due to differential availability of relevant data at the three measurement occasions, the criteria used to classify adolescents as CDF were different at 13, 16 and 18. For example, classifications at 13 were based on parental report, with both parental and child-reported data used at 16, and exclusively child-reported data at 18. We acknowledge the limitation of different reporting methods and that this may lead to slight inconsistencies in the prevalence estimates, with studies reporting differing prevalence estimates depending on whether report was done by parent or child or both.^18^ ^38^ We are confident that our definitions provided a consistent classification of CDF across the different ages, which is supported to some degree by our observed prevalences being consistent with other studies in children and adolescents. At age 16, our definition was based on parent-reported and child-reported data, including a Chalder Fatigue Questionnaire threshold, which has a sensitivity of 82% and a specificity of 86% for CFS/ME.

We included adolescents with both CDF and depressive symptoms. Approximately 30% of children with CFS/ME in specialist services have comorbid depression, and it is not known whether this is a predictor of, or secondary to, CFS/ME.^39^ If depression is secondary to CDF, then by reclassifying those with depression as non-CDF, we would have underestimated prevalence estimates for CDF.

Finally, as we did not have data on whether the adolescents were receiving specialist treatment or not, we are unable to accurately define the ‘natural course’ of CDF during this period. However, data were collected before there was a specialist service available in Bristol, and therefore, it is unlikely that children received specialist treatment.

## Conclusion

In this large cohort of adolescents, the prevalence of CDF (a proxy for clinically diagnosed CFS/ME) increases throughout adolescence, with 2.99% (95% CI 2.24% to 3.75%) of 18 year olds classified as such. Despite this increasing prevalence, we have shown that approximately three quarters of adolescents recovered from a previous classification of CDF, with around 8% of those classified as having CDF at the beginning of adolescence persisting with the condition at both 16 and 18 years. These findings should be interpreted within the limitations of the study, most notably the different definitions of CDF at the three time points.
