# Supplementary material for: Natural course of chronic fatigue syndrome/myalgic encephalomyelitis in adolescents
Source: Arch Dis Child. 2017 Jan 19;102(6):522–8. doi: 10.1136/archdischild-2016-311198 (PMC5466925; doi:10.1136/archdischild-2016-311198)
Supplement: supplementary material [file archdischild-2016-311198supp001.pdf]

Supplementary material

Supplementary table 1 Distribution and amount of data for the imputed variables in those with fatigue data at 18 years

Imputed variable	Distribution statistics for those with complete age 18 fatigue data (n=4290)		
Continuous variables	Mean (SE)/ Median (IQR)	Range	n
FAI_pregnancy	1 (0-1)	0-9	3602
FAI_ 8-10 years	1 (0-2)	0-8	3367
Key stage 2 mark	64.8 (0.004)	12-90.7	3407
Total number year 11 authorised absences	12.5 (5-22)	0-165	3462
Total number year 11 unauthorised absences	0 (0-2)	0-274	3462
SDQ_total_11years	6.1 (0.001)	0-31	3526
Maternal age at delivery	29.3 (0.001)	16-44	4290
Categorical variables			
CDF_13_6months	1.2%		3463
CDF_13_3months	2.6%		3463
CDF_16_6months	1.3%		3304
CDF_16_3months	1.8%		3304
MF0601	3.5%		3577
MF0611	0.3%		3577
MF0612	15.4%		3284
Maternal education:<br>CSE<br>Vocational<br>O level<br>A level<br>Degree	 11.4%<br>7.3%<br>34.2%<br>28.0%<br>19.1%		4150

FAI_preg: family adversity index taken in pregnancy; FAI_8-10years: family adversity index taken in when child aged 8-10years; Key stage 2 mark: calculated from the mean result across English, Maths and Science taken in Year 6 (10 – 11 years) was used; SDQ_total_11 years: total score on Strength and Difficulties Questionnaire at 11 years 8 months; CDF_13_6months: chronic disabling fatigue at 13 using 6 months criterion; CDF_13_3months: chronic disabling fatigue at 13 using 3 months criterion; CDF_16_6months: chronic disabling fatigue at 16 using 6 months criterion; CDF_16_3months: chronic disabling fatigue at 16 using 3 months criterion; MF0601: binary variable indicating early parenthood; MF0611: binary variable indicating major family problems; MF0612: binary variable indicating whether mother experienced any psychopathology CSE: Certificate of Secondary Education; O level: General Certificate of Education (GCE) Ordinary Level; A level: General Certificate of Education (GCE) Advanced Level

Supplementary table 2 Prevalence of chronic disabling fatigue (CDF) at 13 and 16 years

	CDF at 13 years	Prevalence (95% CI)
CDF using 6 month criteria		
Overall		
	Complete	1.13% (0.90% – 1.41%)
	Imputed	1.47% (1.05% – 1.89%)
Males		
	Complete	1.10% (0.80% – 1.51%)
	Imputed	1.65% (0.99% - 2.32%)
Females		
	Complete	1.16% (0.85% -1.59%)
	Imputed	1.28% (0.82% - 1.73%)
CDF using 3 month criteria		
Overall		
	Complete	2.29% (1.96% - 2.68%)
	Imputed	2.54% (2.04% - 3.03%)
Males		
	Complete	2.16% (1.72% - 2.71%)
	Imputed	2.57% (1.81% - 3.33%)
Females		
	Complete	2.42% (1.95% - 3.00%)
	Imputed	2.50% (1.89% - 3.10%)

	CDF at 16 years	Prevalence (95% CI)
CDF using 6 month criteria		
Overall		
	Completed	1.46% (1.18% - 1.80%)
	Imputed	2.22% (1.67% - 2.78%)
Males		
	Complete	1.09% (0.77% - 1.56%)
	Imputed	2.03% (1.21% - 2.85%)
Females		
	Complete	1.79% (1.37% - 2.33%)
	Imputed	2.43% (1.69% - 3.18%)
CDF using 3 month criteria		
Overall		
	Complete	2.02% (1.68% - 2.41%)
	Imputed	2.70% (2.12% - 3.27%)
Males		
	Complete	1.60% (1.20% -2.15%)
	Imputed	2.29% (1.51% - 3.08%)
Females		
	Complete	2.39% (1.90% - 3.00%)
	Imputed	3.13% (2.28% - 3.97%)

*These estimates differ from those previously published using the same ages and from the same cohort (Crawley et al. 2012; Collin et al (in press))

Auxiliary variables:

Family Adversity Index (pregnancy and 8-10 years)

A family adversity index (FAI) was derived from responses to questions asked during gestation and when the child was 8-10 years old about the following family-based factors: age of mother at first pregnancy; housing (1)adequacy; 2)basic amenities; 3)defects/damp/infestations); mother's educational qualifications; financial difficulties; relationship with partner (1)status, 2)affection and aggression, 3)physical and emotional cruelty); family (1)size; 2) problems such as child in care, not with natural mother, on at risk register); social network (1)emotional; 2) practical/financial support); maternal psychopathology (1)anxiety; 2)depression or suicide attempts); substance abuse (drugs or alcohol); and crime (1)in trouble with police; 2)convictions). These ten factors yield an index with a range of 0–18.

Life difficulties

Life difficulties have been reported by parents throughout childhood and adolescence using the Strength and Difficulties Questionnaire (SDQ). This behavioural screening tool asks about 25 attributes, some positive and others negative, divided between 5 sub-scales: emotional symptoms (5 items); conduct problems (5 items); hyperactivity/inattention (5 items); peer relationship problems (5 items); prosocial behaviour (5 items). Responses to each item are scored 0-2 thus allowing each section to be scored from 0-10. A 'total difficulties' score is calculated by adding scores for the first four subscales (excluding prosocial behaviour), yielding a total score with a range of 0 – 40. For this analysis, only the SDQ reported at 140 months (11 years 8 months) was used.

School attendance and academic attainment

The total number of authorized and unauthorized school absences during the Year 11 school year (age 15-16 years) were obtained via linkage to the National Pupil Database (NPD). NPD is a pupil level longitudinal database which matches pupil and school characteristics to pupil level attainment in England (www.gov.uk/government/collections/national-pupil-database). Academic attainment data has also been collected from the NPD. In this analysis, the mean Key Stage 2 (KS2) score, calculated from the results in English, Maths and Science taken in Year 6 (10 – 11 years) was used.

Maternal age at delivery and highest educational attainment

The age of the mother was calculated from the mother's date of birth (obtained at recruitment) for specific time points. Age of mother at delivery was universally collected in the cohort. Maternal highest education qualification was collected from the 'Your pregnancy' questionnaire, which was completed at 32 weeks gestation.

Complete case recovery and persistence results:

Recovery and persistence (6 month criteria): complete case

Of the 52 adolescents classified as having CDF at 13 and with available data to define CDF at 16 years, 11 also presented at 16 with CDF (21.15% 95% CI: 11.88% - 34.81%). This gives a *recovery* rate of 13.67 (95% CI: 9.48 – 17.85) adolescents per year between 13 and 16 years.

In those with complete data, there were no adolescents who appeared to have recovered at 16 and subsequently present at 18 with the condition.

Between 16 and 18 years, 8 out of 43 adolescents (18.60% 95% CI: 9.31% - 33.72%) classified as CDF at 16 and with available data at 18, presented with CDF at 18. This is a recovery rate of 17.5 (95% CI: 11.70 – 23.30) cases per year between 16 and 18 years.

The complete case analysis revealed 2.63% of adolescents (out of those classified as CDF at 13 years) with persistent CDF over the three time points (95% CI 0.64% - 10.21%).

Recovery and persistence (3 month criteria): complete case

Of those with data at both time points 89/105 (84.76% 95% CI: 76.40% – 90.53%) of adolescents who had CDF at 13 did not have CDF at 16. This gives a recovery rate of 29.67 (95% CI: 23.50 – 35.83) adolescents per year between 13 and 16 years.

1.92% (95% CI: 0.25% - 13.18%) of those who had available data at all three time points and appeared to have recovered at 16, had CDF at 18.

Of the 59 adolescents with CDF at 16 and available data at 18, 8 had CDF at 18 (13.56% 95% CI: 6.79% - 25.26%). This is a recovery rate of 25.5 (95% CI: 18.50– 32.50) cases per year between 16 and 18 years.

2.59% of those classified as CDF at 13 years showed persistence throughout adolescent (95% CI 9.68% - 6.78%).

Imputed recovery and persistence results (with CDF defined using a 3 month criterion):

3 month definition: 81.7% (95% CI: 74.5% - 88.9%) of those presenting with CDF at 13 did not subsequently present with the condition at 16. A small percentage (6.48% of (95% CI: 0.44% - 12.5%)) had CDF/ME at 13 and 18 but not 16 years old. Persistence between 16 and 18 years was apparent in 18.6% (95% CI: 8.81% - 28.3%) of adolescents. Finally, 5.55% (95% CI 1.68% - 9.43%) of those classified as CDF at 13 years presented consistently with the condition at 13, 16 and 18 years.

Imputation after reclassifying those with CDF and any depressive symptoms during adolescence to non-CDF, revealed that proportions of those with persistent CDF were very similar between 13-16 years (18.30%; 95% CI: 11.1% -25.5%) but slightly lower between 16-18 years (13.8%; 95% CI: 4.6% - 23.1%), and between all three time points (4.35%; 95% CI: 0.65% - 8.05%).

Supplementary table 3: chronic disabling fatigue (CDF) at later time points by presence of earlier CDF, defined using the 3 month fatigue criterion

		Based on multiple imputation* n/total (%)	Imputed Odds Ratio* (95% CI)
3 month fatigue			
Total sample			
CDF 16			
	CDF at 13^	65/355 (18.3%)	9.5 (5.5 – 16.2)
	No CDF at 13	312/13623 (2.3%)	
Boys			
CDF at age 16	CDF at 13^	32/186 (17.2%)	10.6 (4.8 – 23.2)
	No CDF at 13	134/7034 (1.9%)	
Girls			
CDF at age 16	CDF at 13^	33/169 (19.5%)	8.6 (3.8 – 19.2)
	No CDF at 13	178/6589 (2.7%)	

^based on 3 month fatigue
